# Deep data science to prevent and treat growth faltering in Maya children

**DOI:** 10.1038/ejcn.2016.63

**Published:** 2016-04-20

**Authors:** M I Varela-Silva, B Bogin, J A G Sobral, F Dickinson, S Monserrat-Revillo, the Healthy Birth, Growth, and Development – Knowledge Integration (HBGDki) Initiative

**Affiliations:** 1 School of Sport, Exercise & Health Sciences, Loughborough University, Loughborough, UK; 2 Centro de Investigaciones Educativas, Universidad del Valle de Guatemala, Guatemala City, Guatemala; 3 Department of Human Ecology, Cinvestav-IPN, Mérida, Mexico; 4 Quantitative Sciences, Global Health, Integrated Development, Bill & Melinda Gates Foundation, Seattle, WA, USA

## Abstract

The Maya people are descended from the indigenous inhabitants of southern Mexico, Guatemala and adjacent regions of Central America. In Guatemala, 50% of infants and children are stunted (very low height-for-age), and some rural Maya regions have >70% children stunted. A large, longitudinal, intergenerational database was created to (1) provide deep data to prevent and treat somatic growth faltering and impaired neurocognitive development, (2) detect key dependencies and predictive relations between highly complex, time-varying, and interacting biological and cultural variables and (3) identify targeted multifactorial intervention strategies for field testing and validation. Contributions to this database included data from the Universidad del Valle de Guatemala Longitudinal Study of Child and Adolescent Development, child growth and intergenerational studies among the Maya in Mexico and studies about Maya migrants in the United States.

## Introduction

Childhood growth faltering includes growth stunting and is an indicator of chronic undernutrition.^1, 2, 3^ Stunting increases the risk for disease, impaired neurocognitive development, reduced work capacity and death. Its causes include long-term shortage of energy or essential nutrients, infections, environmental toxins, enteric dysfunction, physical or emotional abuse and psychiatric pathology affecting the endocrine function.^4, 5^ In 2010, there were 171 million stunted children aged <5 years globally,^3^ with 90% living in 39 lower- and middle-income countries. The prevalence of stunting in Asia has decreased since 1985 but has not improved in Africa or Guatemala. In Guatemala, 50% infants and children are stunted, some rural Maya regions have >70% children stunted^6^ and 38% of Maya in rural Guatemala are stunted at birth.^4^

The Maya people descend from the indigenous inhabitants of southeastern Mexico, Guatemala, Belize, San Salvador and Honduras.^7^ They are the largest Native American group (6–7 million people) and show the shortest average height of any non-pygmy human population.^7^

The Maya people speak >30 Mayan languages and Spanish, and practice a mixture of pre-Columbian and Christian traditions. In Guatemala, the Spanish descendants (*ladino*) speak Spanish, practice Christianity and typically deny Maya ancestry. In Mexico, descendants from a mixture of Native American and Spanish ancestry (*mestizo*) typically speak Spanish as their primary language, may also speak a Native American language, and sometimes deny Maya ancestry.

In 2014, the Bill & Melinda Gates Foundation launched the Healthy Birth, Growth and Development-Knowledge Integration (HBGDki) initiative to learn from currently available open-access data and generate novel insights into three interrelated outcomes: preterm birth, growth faltering and impaired neurocognitive development.

Our databases' contributions to this initiative are (1) the Universidad del Valle de Guatemala (UVG) Longitudinal Study of Child and Adolescent Development, a long-term study of child development in Guatemala, (2) child growth and intergenerational studies of Mexican Maya (since the 1980 s) and (3) studies about Maya migrants in the United States, who experienced remarkable increases in average height compared with Maya in Mexico and Guatemala.^1, 8^ The purpose of this report is to present brief descriptions of the contributed data and explain how these may serve to improve healthy birth, growth and development globally.

### The Universidad del Valle de Guatemala Longitudinal Study

The UVG was a large, mixed-longitudinal study of human growth in Latin America and contained 138 000 cross-sectional sets of data (girls and boys aged 4–18 years attending one of seven schools in or near Guatemala City). These sets included ⩾15 000 longitudinal series with 2 to 14 examinations per participant. Between 1953 and 1999, children were assessed annually for height, weight, skinfolds, upper-arm circumference, permanent teeth erupted, hand-grip strength, general cognitive ability and reading performance, and a hand-and-wrist radiograph was taken. Schools were graded for socioeconomic status (SES) based on the annual fee charged. State schools (no fee) in a Maya village outside Guatemala City had the lowest SES. The study included participants with ethnic variation representing the entire population of Guatemala (*ladinos*, Europeans and Maya).

There were >50 publications based on UVG study data, but much available detail was neglected. Analyses were piecemeal, mostly descriptive, and only 144 children of the whole set were investigated for anthropometric, radiographic and cognitive relationships.^9^

Paper records have been converted to electronic files for all anthropometric and cognitive data, as well as for hand-wrist radiographs of all Maya and as many *ladino* and European children as possible within budgetary and time constraints. The radiographs supply information on skeletal age, biological maturity, bone health and residual height growth prediction using automated software (BoneXpert, Visiana, Holte, Denmark).

### Child growth and intergenerational studies among the Maya in Mexico

Growth studies of Maya and non-Maya children in Mexico have been performed for decades.^10^ Previous studies evaluated intergenerational effects on the nutritional status of Maya children in small samples of two and three generation clusters. These studies highlighted the development of the nutritional dual-burden phenomenon (coexistence of stunting with overweight/obesity) in mother–child dyads and individuals.^2^ The impact of the maternal grandmother's childhood health status on fat accumulation in her grandchildren was reported.^10^ Data from these analyses of intergenerational components of health may be combined with others within the HBGDki to gain further insights.

### Studies about Maya migrants in the United States

Studies conducted since 1992 with Maya migrants in Los Angeles and Florida showed a marked increase in average height (11 cm) of the children and reduction in stunting.^1, 10^ Most of these children were born in the United States and had access to clean drinking water, pre- and postnatal health care, food subsidies and a general positive environment despite low-income SES. The change in average height occurred in <10 years but was accompanied by a marked rise in overweight/obesity. This raised new concerns about health outcomes and reinforced the need for further research with greater statistical power. We need to identify and close knowledge gaps about risk factors for stunting and determine the optimal timing in the lifecycle for interventions to ensure maximum coverage, cost-effectiveness and healthiest possible outcomes.

## Conclusion

The longitudinal series in the UVG database may help determine the most sensitive chronological ages and maturational stages for risks of stunting and neurocognitive impairment. The intergenerational data from the Mexican studies may highlight relative contributions of the mother and grandmother on linear and mass growth of children. Limitations of these studies may include a knowledge gap about paternal contributions to child growth and health, and contributing to the HBGDki deep science database may clarify the effect size of these and other knowledge gaps. A deep data knowledge base, with shared information such as this, may enable data-driven, deductive and inductive approaches with minimal bias to understand and improve child somatic growth and neurocognitive development. The sharing of data from the Maya studies may enable robust analyses to determine the interactions of pathophysiologic causal pathways and genetic, epigenetic, environmental, social and demographic factors that directly affect the sensitivity, severity and duration of growth faltering and impaired neurocognitive development.
