# Erratum: Deep data science to prevent and treat growth faltering in Maya children

**DOI:** 10.1038/ejcn.2016.98

**Published:** 2016-06-08

**Authors:** M I Varela-Silva, B Bogin, J A G Sobral, F Dickinson, S Monserrat-Revillo, and the Healthy Birth, Growth, and Development – Knowledge Integration (HBGDki) Initiative

**Keywords:** Malnutrition, Paediatric research, Epidemiology, Databases

**Correction to:** *European Journal of Clinical Nutrition* advance online publication 20 April 2016; doi:10.1038/ejcn.2016.63

**Updated online 8 June 2016:** This article was originally published under NPG's License to Publish, but has now been made available under a CC BY 4.0 license. The PDF and HTML versions of the paper have been modified accordingly.

